# Plastic pollution in agricultural landscapes: an overlooked threat to pollination, biocontrol and food security

**DOI:** 10.1038/s41467-024-52734-3

**Published:** 2024-09-28

**Authors:** Dong Sheng, Siyuan Jing, Xueqing He, Alexandra-Maria Klein, Heinz-R. Köhler, Thomas C. Wanger

**Affiliations:** 1 Sustainable Agricultural Systems & Engineering Lab, School of Engineering, Westlake University, Hangzhou, 310030 China; 2 Key Laboratory of Coastal Environment and Resources of Zhejiang Province, School of Engineering, Westlake University, Hangzhou, 310024 China; 3 College of Environmental and Resource Sciences, Zhejiang University, Hangzhou, 310030 China; 4 Department of Environmental Science and Engineering, Fudan University, Shanghai, 200438 China; 5 Department of Health and Environmental Sciences, School of Science, Xi’an Jiaotong-Liverpool University, Suzhou, 215123 China; 6 Nature Conservation and Landscape Ecology, University of Freiburg, Freiburg, 79106 Germany; 7 Animal Physiological Ecology, University of Tübingen, Tübingen, 72076 Germany; 8 Agroecology, University of Göttingen, Göttingen, 37073 Germany

**Keywords:** Agroecology, Plant ecology, Ecophysiology

## Abstract

Ecosystem services such as pollination and biocontrol may be severely affected by emerging nano/micro-plastics (NMP) pollution. Here, we synthesize the little-known effects of NMP on pollinators and biocontrol agents on the organismal, farm and landscape scale. Ingested NMP trigger organismal changes from gene expression, organ damage to behavior modifications. At the farm and landscape level, NMP will likely amplify synergistic effects with other threats such as pathogens, and may alter floral resource distributions in high NMP concentration areas. Understanding exposure pathways of NMP on pollinators and biocontrol agents is critical to evaluate future risks for agricultural ecosystems and food security.

## Abstract (web summary)

Ecosystem services such as pollination and biocontrol are affected by increasing plastic pollution with potential implications for food security. Here the authors synthesize the little known effects of nano- and microplastics on pollinators and biocontrol agents at the organismal, farm and landscape scale.

## Introduction

Plastic pollution has been increasingly recognized as an emerging threat to human health and the environment^1,2^. The effects of microplastics (diameter ranging from 1 μm to 5 mm, hereafter MP), nanoplastics (diameter smaller than 1 μm, hereafter NP) and their associated chemicals in terrestrial ecosystems have recently moved into focus^3,4^. Publications on nano/micro-plastics (hereafter NMP) effects on the environment have increased over the last decade^5,6^ (Supplementary Fig. 1), showing mostly negative effects of NMP on atmosphere, biosphere, hydrosphere and pedosphere^7–9^. Previous studies have primarily focused on aquatic systems but, recently, NMP pollution in terrestrial systems has received growing attention^3^ (Supplementary Fig. 1). Wind, rain, and runoff facilitate NMP long-range transportation and cause plastic pollution in remote areas far away from pollution sources^9–11^. NMP have various impacts on a wide range of organisms, from microbes and plants to animals and humans^12^ – for instance selectively enriching microbial communities in the “soil plastisphere”^13^, reducing Chlorophyll *b* synthesis in *Bacopa* sp^14^. and inducing oxidative stress in mice^15^. NMP also acts synergistically with other threats^16^ such as neonicotinoids^17^, polycyclic aromatic hydrocarbons (PAHs)^18^ and toxic metals (e.g. Pb)^19^. Current research mainly targets NMP effects on single species/communities, but a synthesis of NMP effects on biodiversity-associated ecosystem services such as pollination and pest control is missing^4,20^, despite these services’ contribution to sustainable food production in diversified farms and landscapes^21,22^.

Arthropod pollinators are essential for the production of 70% of all globally produced food crops^23^, and biocontrol agents provide pest control services worth up to US$ 417 per ha and yr across biomes^24^ with a highly favorable cost-benefit ratio of 1:250^25^. Scale effects enhance pollination and biological pest control and thereby facilitate global food security^26,27^ and the effect of respective pollination and biocontrol is 32% and 23% higher in diversified than non-diversified farms^21^. At the landscape level, bee richness^28^ and biocontrol agents^29^ in diversified systems increased by up to four-fold and 50%, respectively. However, insects as major pollinators and biocontrol agents are globally declining from habitat loss, pathogens and parasites, climate change, and the overuse of pesticides^30,31^. Pollinators and biocontrol agents are likely exposed to and affected by NMP in similar ways to other terrestrial and aquatic organisms^32,33^. For instance, NMP may act synergistically with other threats^16,18^ to pollinators and biocontrol agents^34,35^ because MP can act as carriers and releasers of pollutants and then facilitate organismal ingestion^17,36^. Moreover, plastic pollution for instance from plastic mulching can change the soil structure and properties^37,38^, with implications for plant growths and floral resource distributions in agricultural landscapes^39,40^. However, a synthesis of all known direct and indirect effects of NMP on pollination and biological pest control at the organismal, farm and landscape scale is missing but urgently needed to guide policies and future research activities.

**Fig. 1:**
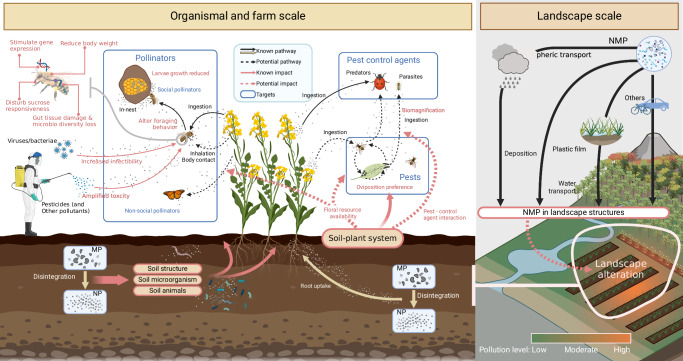
Nano/micro-plastic (NMP) exposure pathways and direct and indirect effects on pollinators, pests and pest control agents at the farm and landscape scale. Known or evidence-supported pathway and effects are shown with solid arrows, and anticipated ones with dashed arrows. The relative importance of these pathways and effects is likely going to differ depending on microplastic types and characteristics such as size and shape. For a detailed description see the main text. MP microplastics, NP nanoplastics, NMP nano/micro-plastics. (Fig. 1, created with BioRender.com, released under a Creative Commons Attribution-NonCommercial-NoDerivs 4.0 International license).

We use a systematic review to quantify all known and potential NMP exposure pathways, as well as the direct and indirect effects of NMP on pollinators and biocontrol agents (Fig. 1). We focus on NMP effects individually and in synergy with other threats from the organism to farm and the landscape scale. After highlighting important research gaps, we close with a research agenda to avoid potentially severe, yet unrecognized threats to global food production.

### Systematic literature review

We use a systematic review to understand the effects of NMP on pollination services and biological pest control (Supplementary Fig. 2 and Supplementary Fig. 3). For pollination services, we searched the Web of Science on April 29th, 2024 with the search string “TS = ((nanoplastic* OR microplastic*) AND (pollinat* OR (bee OR bees) OR honeybee*))”. We found 22 studies out of which 17 were included in our review. Nine research articles reported experiments with NMP on honeybees, five of which focused on NMP effects^41–45^ and the others also considered combined effects with other substances^46–49^. Three studies confirmed honeybees’ environmental exposure to NMP^47,50,51^. The transfer of NMP within bee hives and its threat to honey products were also investigated^50–52^. The remaining five papers were non-quantitative summaries^4,20,53–55^.

For NMP effects on biological pest control, we used the search string “TS = ((nanoplastic* OR microplastic*) AND (“biological pest control” OR “biological control” OR pest OR pests OR pest-control OR “control agent*”))” in Web of Science on April 29^th^, 2024. Of the 24 studies listed, only four were relevant for pest control in agriculture^56–59^ indicating that the topic is largely unexplored. For a summary of all identified effects, please see Table 1.

**Table 1:** Known impacts of NMP on pollinators, pests and biocontrol agents

Target	Type	Impact	Aspect	Effect	Evidence	Referencces
Pollinator *(Apis mellifera)*	MP	Direct	Mortality	No effect or low effect on survival rate	**	^41–43,48,49,52^
			Biomass	Reduce body weight	?	^41,48^
			Food consumption	Less intake of sucrose solution	?	^41–43^
			Behaviors	Disturb sucrose responsiveness	**	^42,45^
				No effect to sucrose preference	**	^42,43^
				Impair learning and memory	*	^45^
			Alimentary system	Damage midgut tissue	*	^47^
				Disrupt gut microbiota community	**	^46,48,49^
			Gene expression	Stimulate expressions related to immunity, detoxification, etc.	**	^47,48^
		Indirect	Infection	More infectious to pathogens	**	^47,49^
			Amplifier of pollutants	More vulnerable to antibiotics	*	^48^
	NP	Direct	Mortality	Reduce survival rate	**	^42,46^
			Biomass	Reduce body weight	*	^46^
			Food consumption	More intake of food	-	^42^
			Behaviors	Disturb PER to sucrose	-	^42^
				No effect to sucrose preference	-	^42^
			Alimentary system	Damage midgut tissue (stronger than MP)	*	^47^
				Induce intestinal dysplasia	*	^46^
				Certain gut microbiota loss	*	^46^
			Gene expression	Stimulate expressions related to immunity, detoxification, etc.	**	^46,47^
		Indirect	Infection	More infectious to pathogens	*	^46^
Pollinator *(Apis cerana)*	MP	Direct	Alimentary system	Damage midgut tissue	*	^47^
			Gene expression	Alter gene expressions	*	^47^
		Indirect	Infection	More infectious to viruses	*	^47^
	NP	Direct	Alimentary system	Damage midgut tissue (stronger than MP)	*	^47^
			Gene expression	Alter gene expressions	*	^47^
Pollinator *(Partamona helleri)*	MP	Direct	Biomass	Increase body weight	*	^44^
			Circulatory System	Change hemocyte counts	*	^44^
			Foraging behavior	Disturb walking behavior	*	^44^
Biocontrol agent *(Hermetia illucens)*	MP	Direct	Larvae	Alter larvae biomass	-	^56^
Biocontrol agent *(Steinernema feltiae)*	NP	Direct	Fitness	Reduced survival, reproduction and pathogenicity	*	^59^
			Physiological	Oxidative stress and mitochondrial dysfunction	*	^59^
Pest *(Bradysia difformis)*	MP	Indirect	Oviposition	Lower oviposition interest to polluted plant-soil systems	*	^57^
Pest *(Culex pipiens* & *Cx tarsalis)*	MP	Direct	Fitness-related	No effects on body size, development and growth rate	*	^58^

**Some supporting literature, i.e., ≥ 2 supporting studies.

*Limited supporting literature, i.e., <2 supporting studies.

-Results not obvious, i.e., the only supporting study used mixtures of NP and MP, or showed mixed effects within the same study;

?Controversial, where conflicting results were provided by different studies.

### NMP exposure pathways to pollinators and biocontrol agents

Pollinators and biocontrol agents are at risk from a plethora of NMP exposure pathways. Plastics accumulate in agricultural landscapes up to ∼2×10^3^ particles/kg in farm soils^38,60^. Direct sources of macroscopic plastic particles include plastic mulch films^38,61^, and protective nets^62^ that result in NMP due to photodegradation, mechanical abration, and biodegradation^63,64^. Indirect plastic inputs result from NMP-polluted sewage sludge^65,66^, fertilizers^67^, compost^38^, irrigation^68^, and manure^69^. Different kinds of NMP occur in the atmosphere as atmospheric fallout, road dust, and even as a substantial component of particulate matter PM_2.5_^9,70,71^ (Fig. 1). Suspended airborne NMP may attach to insects’ surfaces, and the deposited particles found in water and on the inflorescences of flowering plants^72^ may enter insects through ingestion. Additionally, NMP in agricultural soils^73^ may threaten ground-nesting and soil-nesting bees. Pollinators ingest NMP^47^ or collect plastic as nesting materials^74^, and then transfer them into their nests and larvae^52^ (Fig. 1). Certain bee-keeping practices also directly introduce NMP into the nest^50^. All of the above indicates direct exposure of pollinators to NMP pollution.

Biocontrol agents are also likely to ingest NMP while foraging (Fig. 1). Currently, it is difficult to assess whether an increased NMP exposure occurs via bioaccumulation over trophic levels (biomagnification) and, hence, the potential bioaccumulation risk by NMP to pest control agents. This is because the general intracellular uptake of plastics is limited to sizes below 1 µm^75^, which are difficult to quantify analytically^76^ (but see Anbumani and Kakkar^77^). Overall, exposure studies are needed to vastly expand our understanding of the complex NMP exposure pathways on pollinators and biocontrol agents on the organismal but even more so on the farm and landscape level.

### Direct microplastic effects at the organismal level

#### Pollinators

Current evidence from laboratory experiments (Table 1) suggests that MP have limited lethal effects on honeybees in general^41–43^, but reduced the survival rate of newly-emerged worker bees^46^. MP from food and water resources accumulated (i.e., continuous addition to the intestinal lumen without complete removal) in bees’ digestive system, especially in the midgut and hindgut^43,46–49^. Accumulated MP in bee guts can harm tissues (5, 50 μm polystyrene PS-MP)^47^, induce intestinal dysplasia (1100 μm PS-MP)^46^, and alter gut microbiota composition (1, 25, 100 μm PS-MP^46,48^, and 1,10,100 μm PE-MP^49^). Small-sized MP (down to 5 μm) could also enter the respiratory system and accumulate in tracheae^47^, or may penetrate and accumulate in the brain^45^. The circulatory system is affected by early exposure to MP, with a shift of plasmatocytes and prohemocytes (0.5 mm PS-MS, 0.6 mm polyethene terephthalate (PET-MP)^44^. MP also stimulates gene expression related to oxidative stress, the immune system, and detoxification^46,48^.

MP exposure led to a series of behavioral changes (Table 1). For instance, polyethene (PE)-MP intake led to altered food consumption (0.1–100 mg/L, 0.2–9.9 μm) and caused inconsistent proboscis extension responses (hereafter PER) in honeybees^42^. Similarly, Pasquini et al.^45^ reported reduced sucrose responsiveness and impaired learning and memory after PS-MP treatment (0.5–50 mg/L, 4.8−5.8 μm). Moreover, exposure to PS-MP (1–100 mg/L, 27 and 93 μm)^41^ and polyester (PLY)-MP (10–100 mg/L, aerodynamic diameter^78^ = 84 μm)^43^ led to reduced food intake (but see Balzani et al.^42^). Early exposure to PET-MP (12.5 mg/L, 0.6 mm) changed locomotion behaviors of adult bees, including more resting and more interactions^44^. As honeybees showed no preference or avoidance between food and water resources with or without PLY-MP (100 mg/L, 84 μm)^43^, bees may not have the ability to distinguish and, hence, avoid MP in the real environment. Current MP toxicity studies focus on honeybees only and, hence, the potentially different physiological and behavioral effects across different pollinator groups and in different life stages^52,79^ must be urgently addressed to understand the implications for general pollination services^80^.

#### Pests and biocontrol agents

Research on the effects of MP on pests and biocontrol agents is in its infancy, with only four laboratory studies available (Table 1). Rondoni et al.^57^ found that PE-MP (5% of the soil weight, 157 μm) exposure reduces the preference for oviposition on plant leaves in black fungus gnats (*Bradysia difformis*, an important crop pest). Thormeyer and Tseng found no fitness-related effects of PS-MP (200 – 20k items/mL, 4.8–5.8 μm) on *Culex pipiens* and *Culex tarsalis* larvae^58^. For biocontrol agents, Pazmiño et al.^56^ reported inconclusive evidence that MP (5% of feed, 2.12 mm polylactic acid (PLA), 1.71 mm PE, or 1.06 mm PS) exposure could affect the larval development of *Hermetia illucens*, a pest control agent for filth flies^81^. These studies confirm the initial effects of MP exposure on agricultural pests and biocontrol agents. MP biomagnification already is documented in marine systems^82^ and may lead to increased MP exposure for pest predators and stronger impacts on their biological pest control services. Baseline research is urgently needed on direct MP effects on pests and biocontrol agents.

### Direct nanoplastic effects on pollinators and biocontrol agents at the organismal level

NP exhibit novel toxicity effects due to their distinct physical, chemical, and biological properties, such as their shapes and protein/eco-corona^83^. NP can not only cross some biological barriers and act as carriers of toxicants, but also modulate organismal functions such as growth and oxidative cell stress^84,85^. We found only four studies that involved direct NP effects on pollinators (Table 1)^42,46,47,55^. A review of NMP effects on pollinators focuses broadly on physiological aspects, but not on the differences between MP and NP uptake pathways and tissue translocation^55^. Similarly to MP, Wang et al.^46^ showed that oral exposure to NP (100 nm) significantly reduced body weight and survival rate, and induced intestinal dysplasia in honeybees. Deng et al.^47^ found that NP (0.5 μm) was especially harmful (compared with MP) to honeybees by accumulating in the midgut and trachea tissues, and stimulating gene expression. Balzani et al.^42^ used particles between 0.2–9.9 μm, suggesting that their findings on changes in feeding behaviors and mortality might be combined effects of MP and NP (Supplementary Fig. 4). Current studies indicate that smaller size NP tend to have stronger negative effects probably due to increased ability to penetrate biological barriers.

Biocontrol agents and pests are likely threatened by NMP effects similar to those on pollinators, but these former effects are largely speculative at the moment. The only available study suggests that exposure to PS-NP (0.1–1 μm) reduced the survival, reproduction, and pathogenicity of *Steinernema feltiae*, known as an effective biocontrol agent to insect pests (Table 1)^59^.

Research is urgently needed to better understand the effects of both MP and NP on pollinators, but even more so on biocontrol agents and pests. This is, because mechanistically, NP toxicity is complex and often linked to MP exposure. For instance, NP-related oxidative cell stress^84^ can lead to DNA damage, apoptosis, and cell death^86^. Such effects may impair pollinators’ memory, learning, and other behaviors such as reduced reproductive success with implications for pollination services^87,88^. Moreover, the characteristics of nanoparticles suggest different mechanisms between NP and MP, but the available studies for pollinators and pest control agents show no fundamentally different effect from MP and NP exposure. This may be, because the NMP used in current studies vary greatly in terms of doses, shapes (spheres^42^, fibers^43^, and fragments^41^), diameters and chemical components (Supplementary Fig. 4), which will mediate organismal effects. Lastly, NP toxicity may be strongly related to MP exposure level and degradation rate, both at the organismal and landscape level. For example, gut bacteria can degrade MP particles in honey bee hindguts^46^ and thereby exacerbate NP exposure. In agricultural landscapes, MP degradation for instance in soils will increase plant, pollinator and pest control agent exposure to NP, eventually affecting food production and security to an unknown extent.

### Indirect effects of NMP in agricultural landscapes

Agricultural landscape complexity not only mediates pollination and biological pest control services^89,90^ but also affects direct and indirect NMP deposition. Plastic accumulation and retention are likely to be driven by farm and landscape features (Fig. 1)^91^. For instance, individual trees and hedgerows at the farm level prevent runoff and soil erosion, and mediate plastic particle retention. In turn, soil erosion from agricultural landscapes has recently been shown to be a source of NMP in rivers^92^. At the landscape scale, forests and semi-natural habitats can capture fine particulate plastic for instance contained in aerosols^8,93^. The spatial configuration of these structures should correspond to areas with buildup of high NMP concentrations that mediate plastic distribution in natural landscapes^94^, which we refer to as “NMP hotspots” (see landscape scale part in Fig. 1).

#### NMP pollution may amplify other threats

NMP hotspots in agricultural landscapes may amplify other environmental threats to pollinators and pest control agents such as chemical pollution^95,96^ and pathogens^97^. For example, the interaction toxicity of NMP with pesticides^98^ is determined by their physicochemical characteristics such as plastic type, size^99^, surface charge^100^, and concentration^101,102^, which brings indirect risk for terrestrial organisms^70^. Pollinators are known to be affected by pesticides such as neonicotinoids^103^ but also by fungicides^104^ for which exposure may be modified through the catalytic activity of NMP. For instance, the survival rate of honeybees dramatically decreased in a combination treatment with tetracycline and MP as opposed to individual treatments^48^. Mechanistically, NMP can adsorb substances such as PAHs, or persistent organic pollutants (POPs)^105^. This may lead to the accumulation of toxic chemicals on NMP surfaces and potentially a modification of interaction toxicity and overall higher substance concentrations. In the marine environment, adsorbed contaminants on MP surfaces increased toxicity towards mussel embryos^106^. In other examples, however, very high MP concentrations reduced the effectiveness of thiacloprid in chironomid larvae, possibly by diminishing the uptake of the pesticide in the gastrointestinal tract^107^. As pesticides affect both pollinators and biocontrol agents, which in turn are heavily influenced by farm and landscape level effects^108,109^, NMP hotspots may likely mediate these relationships further. Future research should investigate interaction toxicity mechanisms and effects on ecosystem service providers in particular with well-known threats from neonicotinoid pesticides across spatial scales.

Pollinators and biological pest control agents are devastated by pathogenic viruses and bacteria^110,111^ and depend on multi-taxa interactions ranging from invertebrates to microorganisms and fungi^112,113^. In pollination, the impacts of viruses on their hosts are exacerbated by other major stressors such as parasites, poor nutrition, and exposure to chemicals^114,115^. NMP can further enhance the invasion of Israeli Acute Paralysis Virus and *Hafnia alvei* to honeybees by affecting cell membranes (especially NP) and immune systems^46,47,49^. Moreover, microbial communities can colonize plastic particles, which may facilitate the spreading of pathogenic bacteria and fungi, while becoming reservoirs for antibiotic and metal-resistance genes in soils^13^. Overall, NMP hotspots may facilitate unintentional interaction toxicity and higher susceptibility to established and new pathogens in agricultural landscapes, of which most mechanisms and implications urgently require more research.

#### NMP may alter agricultural landscapes and ecosystem services

NMP hotspots may also indirectly affect pollinators and biocontrol agents through changes in agricultural landscapes. It is now well understood how farm and landscape level diversification affects biodiversity-mediated ecosystem services like pollination and biological pest control^89,90,116^. For instance, the distribution and amount of semi-natural habitats modify the abundance and diversity of pollinators and biocontrol agents^117,118^. Mechanistically, patterns are driven by floral resources, nesting opportunities, and chemical inputs^119^, which may be modified from NMP effects on soil properties, plant growth, plant communities^39,120^, reproduction^121^, and microbial communities^122^. The mixed NMP effects on plant growth and yield are highly species-dependent^123^, which can affect plant productivity and community structure^124,125^. Moreover, diverse floral resources can mitigate neonicotinoid and fungicide impacts on wild pollinators^104,126^. Hence, a reduction in floral resources in NMP hotspots may affect colony survival and increase exposure to pesticides or other agricultural chemicals^104^. All of these effects are unstudied but are highly likely to modify the resources available in plant-animal interactions and hence, the effectiveness of pollination and biocontrol services.

### Implications on food security and a way forward

The world is already facing massive impacts on food security due to climate change, pests and diseases affecting yields, and conflicts preventing access to safe and nutritious food^127^. The above effects of NMP on pollination and biocontrol services may further exacerbate food insecurity. At the organismal scale, current evidence suggests that service providers experience sublethal yet profound physiological changes^41–43,56^ that are likely exacerbated by other stressors^46–48,105–107^. Although speculative at this stage, at the farm and landscape scale changes in resource availability (e.g., amount or species assemblages of host plants)^124,125^ or plant-soil system characteristics^39,120^ may restrict distributions of pollinators and biocontrol agents. Modified pollination and pest control services in NMP hotspots may further alter the impacts of climate change on crop yields and distribution. Moreover, a diverse diet requires various species to provide pollination and biocontrol services for a broad range of crop species^128^. Species and variety-dependent NMP effects on pollinators and pest control agents may, therefore, constrain the choice of crop species and varieties in the future. Lastly, the projected surge of plastic waste accumulation (12 million metric tons by 2050) and NMP pollution in the coming decades^1,129^ adds to the current risks of food insecurity^130^, and threatens the stability of global food production.

It is critical to be explicit about the limitations of our review that covers all published research of NMP effects on pollinators and biocontrol agents to then chart a way forward based on a specific research agenda. For instance, the few available field studies and the various doses, types and sizes of NMP used in current lab experiments may not match realistic exposure levels (see Supplementary Fig. S4), which creates uncertainty around the observed effects. Furthermore, NMP effects on a large number of major pollinator and biocontrol agent species such as bumblebees and ladybugs remain unexplored. Necessarily, we had to use evidence based on other realms or from other taxonomic groups to speculate, for example, on potential exposure pathways and interaction toxicity with other pollutants effects based on surrogate literature when direct evidence is limited.

In addition to acknowledging plastic pollution as a key concern for biodiversity and associated services^131^, we advocate for research on how diversified agricultural landscapes^132^ mediate the tradeoff between pollinator and pest control benefits and accumulation effects at “NMP hotspots” to ensure long-term maintenance of crop yields and food security^133^. Future research should target the development and refinement of methods that can be applied in laboratory, semi-field, and field studies to address global food security implications (Box 1). Additional funding should be allocated specifically to understand NMP effects across scales on biodiversity-associated ecosystem services such as pollination and biological pest control in pursuit of the Global Biodiversity Framework’s roadmap for biodiversity conservation^131^ and a food-secure future.

Box 1: Future research directions to address urgent knowledge gaps of NMP effects on pollinators and pest control agents with implications for food security

1. **Develop methods to detect NMP in environmental samples**. Despite more effective methods for NMP detection in environmental samples being continuously developed^76,134^, the detection of small-sized plastics ( < 1 μm) in real environments and organisms remains a critical bottleneck. Moreover, methods to automate sample preparation and analysis are currently limiting NMP research, especially in terrestrial systems, where samples are comprised of an organic and much more complicated matrix than in water^134,135^. In addition, it becomes increasingly clear that plastic pollution exhibits less acute and more chronic effects, which require standard methods to effectively track NP and their interaction effects in pollinators, biocontrol agents, and more broadly ecosystem service-providing insect communities.
2. **Conduct ecosystem ecotoxicology studies of NMP**. Laboratory studies have shown the ecotoxicity of NMP on different organisms, but only to a limited extent on pollinators and biocontrol agents. In addition, concentrations used in current studies are often likely too high compared to largely unknown real-field NMP exposure, similar to the aquatic environment^75^. More systematic perspectives involving various ecosystem agents should be adopted, for instance, the “novel epidemiology” concept^136^, whereby a plant-pollinator-pathogen network is used to analyze plant-pollinator extinction. As NMP properties change greatly due to weathering and chemical degradation, different NMP properties across realistic environmental concentrations must be investigated on commercial (e.g., honey bees) and wild pollinators (e.g., solitary bees and hoverflies acting as pollinators and pest-control agents) in controlled environmental conditions. In addition, the newly developed methods above should identify realistic NMP concentrations to be used in semi-field and field effect studies on pollinators and biocontrol agents’ acute and chronic lethal and sublethal toxicity and the effects on behaviour and their ecosystem services on the semi-field to field scale. Specifically, NMP metabolites, leachate, and interaction toxicity require more attention.
3. **Understand NMP impact mitigation**. In urban environments, PM_2.5_ contains up to 13.2% fine particulate plastic and can be mitigated through green wall structures and planted roofs, which would also reduce exposure to pollinators. Moreover, small trees and shrubs can also improve air quality in streets^137,138^. Designing vegetation barriers for NMP transfer depends on the choice of plant species and composition and their spatial configuration^139,140^. This could be integrated into systematic conservation planning^141^ practices to conserve endangered pollinators and biocontrol agents. However, understanding the role of landscape heterogeneity in potentially mitigating plastic but also chemical pollution in agricultural landscapes across scales is an emerging area of research.

## Supplementary information

Supplementary Information

## References

[1] Borrelle, S. B. et al. Predicted growth in plastic waste exceeds efforts to mitigate plastic pollution. *Science* **369**, 1515–1518 (2020). 32943526 10.1126/science.aba3656

[2] Vethaak, A. D. & Legler, J. Microplastics and human health. *Science* **371**, 672–674 (2021). 33574197 10.1126/science.abe5041

[3] Rillig, M. C. & Lehmann, A. Microplastic in terrestrial ecosystems. *Science* **368**, 1430–1431 (2020). 32587009 10.1126/science.abb5979 PMC7115994

[4] Baho, D. L., Bundschuh, M. & Futter, M. N. Microplastics in terrestrial ecosystems: Moving beyond the state of the art to minimize the risk of ecological surprise. *Glob. Change Biol.* **27**, 3969–3986 (2021). 10.1111/gcb.15724 34042229

[5] Akdogan, Z. & Guven, B. Microplastics in the environment: A critical review of current understanding and identification of future research needs. *Environ. Pollut.* **254**, 113011 (2019). 31404735 10.1016/j.envpol.2019.113011

[6] Hale, R. C., Seeley, M. E., La Guardia, M. J., Mai, L. & Zeng, E. Y. A global perspective on microplastics. *J. Geophys. Res. Oceans* **125**, (2020).

[7] MacLeod, M., Arp, H. P. H., Tekman, M. B. & Jahnke, A. The global threat from plastic pollution. *Science* **373**, 61–65 (2021). 34210878 10.1126/science.abg5433

[8] Aeschlimann, M., Li, G., Kanji, Z. A. & Mitrano, D. M. Potential impacts of atmospheric microplastics and nanoplastics on cloud formation processes. *Nat. Geosci.* **15**, 967–975 (2022). 36532143 10.1038/s41561-022-01051-9 PMC7613933

[9] Chen, Y. et al. Quantification and characterization of fine plastic particles as considerable components in atmospheric fine particles.*Environ. Sci. Technol.* **58**, 4691–4703 (2024). 38323401 10.1021/acs.est.3c06832

[10] Dong, H. et al. Microplastics in a remote lake basin of the tibetan plateau: impacts of atmospheric transport and glacial melting. *Environ. Sci. Technol*. acs.est.1c03227 10.1021/acs.est.1c03227 (2021). 10.1021/acs.est.1c03227 34524792

[11] Kane, I. A. et al. Seafloor microplastic hotspots controlled by deep-sea circulation. *Science* **368**, 1140–1145 (2020). 32354839 10.1126/science.aba5899

[12] Toussaint, B. et al. Review of micro- and nanoplastic contamination in the food chain. *Food Addit. Contam. Part A* **36**, 639–673 (2019). 10.1080/19440049.2019.1583381 30985273

[13] Rillig, M. C., Kim, S. W. & Zhu, Y.-G. The soil plastisphere. *Nat. Rev. Microbiol*. 10.1038/s41579-023-00967-2 (2023). 10.1038/s41579-023-00967-2 PMC7615554 37697003

[14] Yu, H. et al. Microplastic residues in wetland ecosystems: Do they truly threaten the plant-microbe-soil system? *Environ. Int.* **156**, 106708 (2021). 34153891 10.1016/j.envint.2021.106708

[15] Wang, S. et al. Polystyrene microplastics affect learning and memory in mice by inducing oxidative stress and decreasing the level of acetylcholine. *Food Chem. Toxicol.* **162**, 112904 (2022). 35257813 10.1016/j.fct.2022.112904

[16] Brook, B., Sodhi, N. & Bradshaw, C. Synergies among extinction drivers under global change. *Trends Ecol. Evol.* **23**, 453–460 (2008). 18582986 10.1016/j.tree.2008.03.011

[17] Zhou, S. et al. Effects of neonicotinoid insecticides on transport of non-degradable agricultural film microplastics. *Water Res* **236**, 119939 (2023). 37054611 10.1016/j.watres.2023.119939

[18] Trevisan, R., Voy, C., Chen, S. & Di Giulio, R. T. Nanoplastics decrease the toxicity of a complex PAH mixture but impair mitochondrial energy production in developing zebrafish. *Environ. Sci. Technol.* **53**, 8405–8415 (2019). 31259535 10.1021/acs.est.9b02003 PMC6660138

[19] Liu, S. et al. Microplastics as a vehicle of heavy metals in aquatic environments: a review of adsorption factors, mechanisms, and biological effects. *J. Environ. Manag.* **302**, 113995 (2022). 10.1016/j.jenvman.2021.113995 34700080

[20] Souza Machado, A. A., Kloas, W., Zarfl, C., Hempel, S. & Rillig, M. C. Microplastics as an emerging threat to terrestrial ecosystems. *Glob. Change Biol.* **24**, 1405–1416 (2018). 10.1111/gcb.14020 PMC5834940 29245177

[21] Tamburini, G. et al. Agricultural diversification promotes multiple ecosystem services without compromising yield. *Sci. Adv.* **6**, eaba1715 (2020). 33148637 10.1126/sciadv.aba1715 PMC7673676

[22] Wanger, T. C. et al. Integrating agroecological production in a robust post-2020 Global Biodiversity Framework. *Nat. Ecol. Evol.* **4**, 1150–1152 (2020). 32690908 10.1038/s41559-020-1262-y

[23] Klein, A.-M. et al. Importance of pollinators in changing landscapes for world crops. *Proc. R. Soc. B Biol. Sci.* **274**, 303–313 (2007). 10.1098/rspb.2006.3721 PMC1702377 17164193

[24] Costanza, R. et al. The value of the world’s ecosystem services and natural capital. *Nature* **387**, 253–260 (1997).

[25] Bale, J. S., van Lenteren, J. C. & Bigler, F. Biological control and sustainable food production. *Philos. Trans. R. Soc. B Biol. Sci.* **363**, 761–776 (2008). 10.1098/rstb.2007.2182 PMC2610108 17827110

[26] Tscharntke, T., Grass, I., Wanger, T. C., Westphal, C. & Batáry, P. Beyond organic farming – harnessing biodiversity-friendly landscapes. *Trends Ecol. Evol.* **36**, 919–930 (2021). 34362590 10.1016/j.tree.2021.06.010

[27] Requier, F. et al. Bee and non-bee pollinator importance for local food security. *Trends Ecol. Evol.* **38**, 196–205 (2023). 36503679 10.1016/j.tree.2022.10.006

[28] Kennedy, C. M. et al. A global quantitative synthesis of local and landscape effects on wild bee pollinators in agroecosystems. *Ecol. Lett.* **16**, 584–599 (2013). 23489285 10.1111/ele.12082

[29] Chaplin-Kramer, R., O’Rourke, M. E., Blitzer, E. J. & Kremen, C. A meta-analysis of crop pest and natural enemy response to landscape complexity: Pest and natural enemy response to landscape complexity. *Ecol. Lett.* **14**, 922–932 (2011). 21707902 10.1111/j.1461-0248.2011.01642.x

[30] Rumohr, Q. et al. Drivers and pressures behind insect decline in Central and Western Europe based on long-term monitoring data. *PLOS ONE* **18**, e0289565 (2023). 37611013 10.1371/journal.pone.0289565 PMC10446172

[31] Potts, S. G. et al. Global pollinator declines: trends, impacts and drivers. *Trends Ecol. Evol.* **25**, 345–353 (2010). 20188434 10.1016/j.tree.2010.01.007

[32] Oliveira, M., Ameixa, O. M. C. C. & Soares, A. M. V. M. Are ecosystem services provided by insects “bugged” by micro (nano)plastics? *TrAC Trends Anal*. *Chem* **113**, 317–320 (2019).

[33] Shen, J., Liang, B. & Jin, H. The impact of microplastics on insect physiology and the indication of hormesis. *TrAC Trends Anal. Chem.* **165**, 117130 (2023).

[34] Lorenz, C. S. et al. Nano-sized Al_2_O_3_ reduces acute toxic effects of thiacloprid on the non-biting midge Chironomus riparius. *PLOS ONE* **12**, e0176356 (2017). 28464012 10.1371/journal.pone.0176356 PMC5413047

[35] Mammo, F. K. et al. Microplastics in the environment: Interactions with microbes and chemical contaminants. *Sci. Total Environ.* **743**, 140518 (2020). 32653705 10.1016/j.scitotenv.2020.140518

[36] Jeong, C.-B. et al. Nanoplastic ingestion enhances toxicity of persistent organic pollutants (pops) in the monogonont rotifer brachionus koreanus via multixenobiotic resistance (mxr) disruption. *Environ. Sci. Technol.* **52**, 11411–11418 (2018). 30192528 10.1021/acs.est.8b03211

[37] Amare, G. & Desta, B. Coloured plastic mulches: impact on soil properties and crop productivity. *Chem. Biol. Technol. Agric.* **8**, 4 (2021).

[38] Van Schothorst, B., Beriot, N., Huerta Lwanga, E. & Geissen, V. Sources of light density microplastic related to two agricultural practices: the use of compost and plastic mulch. *Environments* **8**, 36 (2021).

[39] Huang, D. et al. Research progress of microplastics in soil-plant system: ecological effects and potential risks. *Sci. Total Environ.* **812**, 151487 (2022). 34742990 10.1016/j.scitotenv.2021.151487

[40] Rillig, M. C., Lehmann, A., De Souza Machado, A. A. & Yang, G. Microplastic effects on plants. *N. Phytol.* **223**, 1066–1070 (2019). 10.1111/nph.15794 30883812

[41] Al Naggar, Y. et al. Chronic exposure to polystyrene microplastic fragments has no effect on honey bee survival, but reduces feeding rate and body weight. *Toxics* **11**, 100 (2023). 36850975 10.3390/toxics11020100 PMC9963634

[42] Balzani, P. et al. Acute and chronic ingestion of polyethylene (PE) microplastics has mild effects on honey bee health and cognition. *Environ. Pollut.* **305**, 119318 (2022). 35447255 10.1016/j.envpol.2022.119318

[43] Buteler, M. et al. Acute toxicity of microplastic fibers to honeybees and effects on foraging behavior. *Sci. Total Environ.* **822**, 153320 (2022). 35074382 10.1016/j.scitotenv.2022.153320

[44] Viana, T. A. et al. Ingesting microplastics or nanometals during development harms the tropical pollinator Partamona helleri (Apinae: Meliponini). *Sci. Total Environ.* **893**, 164790 (2023). 37321503 10.1016/j.scitotenv.2023.164790

[45] Pasquini, E. et al. Microplastics reach the brain and interfere with honey bee cognition. *Sci. Total Environ.* **912**, 169362 (2024). 38128669 10.1016/j.scitotenv.2023.169362

[46] Wang, K. et al. Nano- and micro-polystyrene plastics disturb gut microbiota and intestinal immune system in honeybee. *Sci. Total Environ.* **842**, 156819 (2022). 35738383 10.1016/j.scitotenv.2022.156819

[47] Deng, Y. et al. Microplastic polystyrene ingestion promotes the susceptibility of honeybee to viral infection. *Environ. Sci. Technol.* **55**, 11680–11692 (2021). 34374532 10.1021/acs.est.1c01619

[48] Wang, K. et al. Gut microbiota protects honey bees (Apis mellifera L.) against polystyrene microplastics exposure risks. *J. Hazard. Mater.* **402**, 123828 (2021). 33254809 10.1016/j.jhazmat.2020.123828

[49] Zhu, L., Wang, K., Wu, X., Zheng, H. & Liao, X. Association of specific gut microbiota with polyethylene microplastics caused gut dysbiosis and increased susceptibility to opportunistic pathogens in honeybees. *Sci. Total Environ.* **918**, 170642 (2024). 38320694 10.1016/j.scitotenv.2024.170642

[50] Buteler, M., Villalobos, E., Alma, A. M., Silva, L. & Tomba, J. P. Management practice for small hive beetle as a source of microplastic contamination in honey and honeybee colonies. *Environ. Pollut.* **334**, 122151 (2023). 37437762 10.1016/j.envpol.2023.122151

[51] Edo, C. et al. Honeybees as active samplers for microplastics. *Sci. Total Environ.* **767**, 144481 (2021). 33450591 10.1016/j.scitotenv.2020.144481

[52] Alma, A. M., De Groot, G. S. & Buteler, M. Microplastics incorporated by honeybees from food are transferred to honey, wax and larvae. *Environ. Pollut.* **320**, 121078 (2023). 36642174 10.1016/j.envpol.2023.121078

[53] Al Naggar, Y. et al. Are honey bees at risk from microplastics? *Toxics* **9**, 109 (2021). 34063384 10.3390/toxics9050109 PMC8156821

[54] Lamas, M., Rodrigues, F., Amaral, M. H., Delerue-Matos, C. & Fernandes, V. C. Contaminant cocktails of high concern in honey: Challenges, QuEChERS extraction and levels. *Separations* **10**, 142 (2023).

[55] Shah, S., Ilyas, M., Li, R., Yang, J. & Yang, F.-L. Microplastics and nanoplastics effects on plant–pollinator interaction and pollination biology. *Environ. Sci. Technol.* **57**, 6415–6424 (2023). 37068375 10.1021/acs.est.2c07733

[56] Pazmiño, M. F., Del Hierro, A. G. & Flores, F. J. Genetic diversity and organic waste degrading capacity of *Hermetia illucens* from the evergreen forest of the Equatorial Choco lowland. *PeerJ* **11**, e14798 (2023). 36755868 10.7717/peerj.14798 PMC9901308

[57] Rondoni, G., Chierici, E., Agnelli, A. & Conti, E. Microplastics alter behavioural responses of an insect herbivore to a plant-soil system. *Sci. Total Environ.* **787**, 147716 (2021). 10.1016/j.dib.2021.107297 PMC8379301 34458524

[58] Thormeyer, M. & Tseng, M. No effect of realistic microplastic exposure on growth and development of wild-caught culex (diptera: culicidae) mosquitoes. *J. Med. Entomol.* **60**, 604–607 (2023). 36798997 10.1093/jme/tjad014

[59] Li, M. et al. Toxicological impacts of microplastics on virulence, reproduction and physiological process of entomopathogenic nematodes. *Ecotoxicol. Environ. Saf.* **273**, 116153 (2024). 38422790 10.1016/j.ecoenv.2024.116153

[60] Beriot, N., Peek, J., Zornoza, R., Geissen, V. & Huerta Lwanga, E. Low density-microplastics detected in sheep faeces and soil: A case study from the intensive vegetable farming in Southeast Spain. *Sci. Total Environ.* **755**, 142653 (2021). 33069476 10.1016/j.scitotenv.2020.142653

[61] Qi, R., Jones, D. L., Li, Z., Liu, Q. & Yan, C. Behavior of microplastics and plastic film residues in the soil environment: A critical review. *Sci. Total Environ.* **703**, 134722 (2020). 31767311 10.1016/j.scitotenv.2019.134722

[62] Piehl, S. et al. Identification and quantification of macro- and microplastics on an agricultural farmland. *Sci. Rep.* **8**, 17950 (2018). 30560873 10.1038/s41598-018-36172-y PMC6299006

[63] Song, Y. K. et al. Combined effects of uv exposure duration and mechanical abrasion on microplastic fragmentation by polymer type. *Environ. Sci. Technol.* **51**, 4368–4376 (2017). 28249388 10.1021/acs.est.6b06155

[64] Omidoyin, K. C. & Jho, E. H. Effect of microplastics on soil microbial community and microbial degradation of microplastics in soil: A review. *Environ. Eng. Res.* **28**, 220716–0 (2023).

[65] Corradini, F. et al. Evidence of microplastic accumulation in agricultural soils from sewage sludge disposal. *Sci. Total Environ.* **671**, 411–420 (2019). 30933797 10.1016/j.scitotenv.2019.03.368

[66] Ziajahromi, S., Neale, P. A., Rintoul, L. & Leusch, F. D. L. Wastewater treatment plants as a pathway for microplastics: Development of a new approach to sample wastewater-based microplastics. *Water Res* **112**, 93–99 (2017). 28160700 10.1016/j.watres.2017.01.042

[67] Weithmann, N. et al. Organic fertilizer as a vehicle for the entry of microplastic into the environment. *Sci. Adv.* **4**, eaap8060 (2018). 29632891 10.1126/sciadv.aap8060 PMC5884690

[68] Zhou, B. et al. Microplastics in agricultural soils on the coastal plain of Hangzhou Bay, east China: Multiple sources other than plastic mulching film. *J. Hazard. Mater.* **388**, 121814 (2020). 31843412 10.1016/j.jhazmat.2019.121814

[69] Yang, J. et al. Abundance and morphology of microplastics in an agricultural soil following long-term repeated application of pig manure. *Environ. Pollut.* **272**, 116028 (2021). 33199067 10.1016/j.envpol.2020.116028

[70] Huang, Y., Qing, X., Wang, W., Han, G. & Wang, J. Mini-review on current studies of airborne microplastics: Analytical methods, occurrence, sources, fate and potential risk to human beings. *TrAC Trends Anal. Chem.* **125**, 115821 (2020).

[71] Klöckner, P., Seiwert, B., Wagner, S. & Reemtsma, T. Organic markers of tire and road wear particles in sediments and soils: transformation products of major antiozonants as promising candidates. *Environ. Sci. Technol.* **55**, 11723–11732 (2021). 34488356 10.1021/acs.est.1c02723

[72] Liebezeit, G. & Liebezeit, E. Non-pollen particulates in honey and sugar. *Food Addit. Contam. Part A* **30**, 2136–2140 (2013). 10.1080/19440049.2013.843025 24160778

[73] Möller, J. N., Löder, M. G. J. & Laforsch, C. Finding microplastics in soils: a review of analytical methods. *Environ. Sci. Technol.* **54**, 2078–2090 (2020). 31999440 10.1021/acs.est.9b04618

[74] Allasino, M. L., Marrero, H. J., Dorado, J. & Torretta, J. P. Scientific note: first global report of a bee nest built only with plastic. *Apidologie* **50**, 230–233 (2019).

[75] Triebskorn, R. et al. Relevance of nano- and microplastics for freshwater ecosystems: A critical review. *TrAC Trends Anal. Chem.* **110**, 375–392 (2019).

[76] Ivleva, N. P. Chemical analysis of microplastics and nanoplastics: challenges, advanced methods, and perspectives. *Chem. Rev.* **121**, 11886–11936 (2021). 34436873 10.1021/acs.chemrev.1c00178

[77] Anbumani, S. & Kakkar, P. Ecotoxicological effects of microplastics on biota: a review. *Environ. Sci. Pollut. Res.* **25**, 14373–14396 (2018). 10.1007/s11356-018-1999-x 29680884

[78] Prodi, V., De Zaiacomo, T., Hochrainer, D. & Spurny, K. Fibre collection and measurement with the inertial spectrometer. *J. Aerosol Sci.* **13**, 49–58 (1982).

[79] Diaz-Basantes, M. F., Conesa, J. A. & Fullana, A. Microplastics in honey, beer, milk and refreshments in Ecuador as emerging contaminants. *Sustainability* **12**, 5514 (2020).

[80] Potts, S. G. et al. Safeguarding pollinators and their values to human well-being. *Nature* **540**, 220–229 (2016). 27894123 10.1038/nature20588

[81] Miranda, C. D., Cammack, J. A. & Tomberlin, J. K. Interspecific competition between the house fly, Musca domestica L. (Diptera: Muscidae) and black soldier fly, Hermetia illucens (L.) (Diptera: Stratiomyidae) when reared on poultry manure. *Insects* **10**, 440 (2019). 31817890 10.3390/insects10120440 PMC6956010

[82] Miller, M. E., Motti, C. A., Hamann, M. & Kroon, F. J. Assessment of microplastic bioconcentration, bioaccumulation and biomagnification in a simple coral reef food web. *Sci. Total Environ.* **858**, 159615 (2023). 36309288 10.1016/j.scitotenv.2022.159615

[83] Junaid, M. & Wang, J. Interaction of nanoplastics with extracellular polymeric substances (EPS) in the aquatic environment: A special reference to eco-corona formation and associated impacts. *Water Res* **201**, 117319 (2021). 34130084 10.1016/j.watres.2021.117319

[84] Sun, X. et al. Toxicities of polystyrene nano- and microplastics toward marine bacterium Halomonas alkaliphila. *Sci. Total Environ.* **642**, 1378–1385 (2018). 30045518 10.1016/j.scitotenv.2018.06.141

[85] Jiang, W., Kim, B. Y. S., Rutka, J. T. & Chan, W. C. W. Nanoparticle-mediated cellular response is size-dependent. *Nat. Nanotechnol.* **3**, 145–150 (2008). 18654486 10.1038/nnano.2008.30

[86] Prüst, M., Meijer, J. & Westerink, R. H. S. The plastic brain: neurotoxicity of micro- and nanoplastics. *Part. Fibre Toxicol.* **17**, 24 (2020). 32513186 10.1186/s12989-020-00358-y PMC7282048

[87] Farooqui, T. Iron-induced oxidative stress modulates olfactory learning and memory in honeybees. *Behav. Neurosci.* **122**, 433–447 (2008). 18410182 10.1037/0735-7044.122.2.433

[88] Massaad, C. A. & Klann, E. Reactive oxygen species in the regulation of synaptic plasticity and memory. *Antioxid. Redox Signal.* **14**, 2013–2054 (2011). 20649473 10.1089/ars.2010.3208 PMC3078504

[89] Haan, N. L., Zhang, Y. & Landis, D. A. Predicting landscape configuration effects on agricultural pest suppression. *Trends Ecol. Evol.* **35**, 175–186 (2020). 31699410 10.1016/j.tree.2019.10.003

[90] Toledo-Hernández, M. et al. Landscape and farm-level management for conservation of potential pollinators in Indonesian cocoa agroforests. *Biol. Conserv.* **257**, 109106 (2021).

[91] Dikareva, N. & Simon, K. S. Microplastic pollution in streams spanning an urbanisation gradient. *Environ. Pollut.* **250**, 292–299 (2019). 31003141 10.1016/j.envpol.2019.03.105

[92] Wang, Y. et al. Soil erosion is a major drive for nano & micro-plastics to enter riverine systems from cultivated land. *Water Res* **256**, 121597 (2024). 38614030 10.1016/j.watres.2024.121597

[93] Allen, S. et al. Atmospheric transport and deposition of microplastics in a remote mountain catchment. *Nat. Geosci.* **12**, 339–344 (2019).

[94] Lwanga, E. H. et al. Microplastic appraisal of soil, water, ditch sediment and airborne dust: The case of agricultural systems. *Environ. Pollut.* **316**, 120513 (2023). 36374801 10.1016/j.envpol.2022.120513

[95] Almeida, R. A., Lemmens, P., De Meester, L. & Brans, K. I. Differential local genetic adaptation to pesticide use in organic and conventional agriculture in an aquatic non-target species. *Proc. R. Soc. B Biol. Sci.* **288**, 20211903 (2021). 10.1098/rspb.2021.1903 PMC8596010 34784768

[96] Vanbergen, A. J. A cocktail of pesticides, parasites and hunger leaves bees down and out. *Nature* **596**, 351–352 (2021). 34349271 10.1038/d41586-021-02079-4

[97] Vurro, M., Bonciani, B. & Vannacci, G. Emerging infectious diseases of crop plants in developing countries: impact on agriculture and socio-economic consequences. *Food Secur* **2**, 113–132 (2010).

[98] Peña, A., Rodríguez-Liébana, J. A. & Delgado-Moreno, L. Interactions of microplastics with pesticides in soils and their ecotoxicological implications. *Agronomy* **13**, 701 (2023).

[99] Jeong, C.-B. et al. Microplastic size-dependent toxicity, oxidative stress induction, and p-JNK and p-p38 activation in the monogonont rotifer (Brachionus koreanus). *Environ. Sci. Technol.* **50**, 8849–8857 (2016). 27438693 10.1021/acs.est.6b01441

[100] Sun, X.-D. et al. Differentially charged nanoplastics demonstrate distinct accumulation in Arabidopsis thaliana. *Nat. Nanotechnol.* **15**, 755–760 (2020). 32572228 10.1038/s41565-020-0707-4

[101] Li, Z. et al. Combined effect of polystyrene microplastics and dibutyl phthalate on the microalgae Chlorella pyrenoidosa. *Environ. Pollut.* **257**, 113604 (2020). 31761578 10.1016/j.envpol.2019.113604

[102] Rowenczyk, L. et al. Microstructure characterization of oceanic polyethylene debris. *Environ. Sci. Technol.* **54**, 4102–4109 (2020). 32150389 10.1021/acs.est.9b07061

[103] Blacquière, T., Smagghe, G., Van Gestel, C. A. M. & Mommaerts, V. Neonicotinoids in bees: a review on concentrations, side-effects and risk assessment. *Ecotoxicology* **21**, 973–992 (2012). 22350105 10.1007/s10646-012-0863-x PMC3338325

[104] Wintermantel, D. et al. Flowering resources modulate the sensitivity of bumblebees to a common fungicide. *Sci. Total Environ.* **829**, 154450 (2022). 35276144 10.1016/j.scitotenv.2022.154450

[105] Zhang, P., Huang, P., Sun, H., Ma, J. & Li, B. The structure of agricultural microplastics (PT, PU and UF) and their sorption capacities for PAHs and PHE derivates under various salinity and oxidation treatments. *Environ. Pollut.* **257**, 113525 (2020). 31761592 10.1016/j.envpol.2019.113525

[106] Gray, A. D. & Weinstein, J. E. Size- and shape-dependent effects of microplastic particles on adult daggerblade grass shrimp (*Palaemonetes pugio*): Uptake and retention of microplastics in grass shrimp. *Environ. Toxicol. Chem.* **36**, 3074–3080 (2017). 28594093 10.1002/etc.3881

[107] Krais, S. et al. Polystyrene microplastics modulate the toxicity of the hydrophilic insecticide Thiacloprid for Chironomid larvae and also influence their burrowing behavior. *Microplastics* **1**, 505–519 (2022).

[108] Bloom, E. H. et al. Synergism between local‐ and landscape‐level pesticides reduces wild bee floral visitation in pollinator‐dependent crops. *J. Appl. Ecol.* **58**, 1187–1198 (2021).

[109] Ricci, B. et al. Local pesticide use intensity conditions landscape effects on biological pest control. *Proc. R. Soc. B Biol. Sci.* **286**, 20182898 (2019). 10.1098/rspb.2018.2898 PMC6571472 31164058

[110] DeGrandi-Hoffman, G., Chen, Y., Huang, E. & Huang, M. H. The effect of diet on protein concentration, hypopharyngeal gland development and virus load in worker honey bees (Apis mellifera L.). *J. Insect Physiol.* **56**, 1184–1191 (2010). 20346950 10.1016/j.jinsphys.2010.03.017

[111] Wu, M. et al. Inhibitory effect of gut bacteria from the Japanese honey bee, Apis cerana japonica, against Melissococcus plutonius, the causal agent of European foulbrood disease. *J. Insect Sci.* **14**, 129 (2014). 25368073 10.1093/jis/14.1.129 PMC4222316

[112] Van Lenteren, J. C., Bolckmans, K., Köhl, J., Ravensberg, W. J. & Urbaneja, A. Biological control using invertebrates and microorganisms: plenty of new opportunities. *BioControl* **63**, 39–59 (2018).

[113] Fisher, M. C. et al. Emerging fungal threats to animal, plant and ecosystem health. *Nature* **484**, 186–194 (2012). 22498624 10.1038/nature10947 PMC3821985

[114] Grozinger, C. M. & Flenniken, M. L. Bee viruses: Ecology, pathogenicity, and impacts. *Annu. Rev. Entomol.* **64**, 205–226 (2019). 30629896 10.1146/annurev-ento-011118-111942

[115] Annoscia, D. et al. Neonicotinoid Clothianidin reduces honey bee immune response and contributes to Varroa mite proliferation. *Nat. Commun.* **11**, 5887 (2020). 33208729 10.1038/s41467-020-19715-8 PMC7675992

[116] Mitchell, M. G. E. et al. Reframing landscape fragmentation’s effects on ecosystem services. *Trends Ecol. Evol.* **30**, 190–198 (2015). 25716547 10.1016/j.tree.2015.01.011

[117] Sánchez-Bayo, F. & Wyckhuys, K. A. G. Worldwide decline of the entomofauna: a review of its drivers. *Biol. Conserv.* **232**, 8–27 (2019).

[118] Tscharntke, T. et al. When natural habitat fails to enhance biological pest control – Five hypotheses. *Biol. Conserv.* **204**, 449–458 (2016).

[119] Tscharntke, T., Grass, I., Wanger, T. C., Westphal, C. & Batáry, P. Restoring biodiversity needs more than reducing pesticides. *Trends Ecol. Evol.* **37**, 115–116 (2022). 34922780 10.1016/j.tree.2021.11.009

[120] Zang, H. et al. Microplastics in the agroecosystem: Are they an emerging threat to the plant-soil system? *Soil Biol. Biochem.* **148**, 107926 (2020).

[121] Carvallo, G. O. & Muñoz-Michea, V. Polypropylene fragments block pollen–pistil interactions and reduce seed production in a monkeyflower species. *Environ. Sci. Technol. Lett*. acs.estlett.4c00034 10.1021/acs.estlett.4c00034 (2024).

[122] Wang, F., Wang, Q., Adams, C. A., Sun, Y. & Zhang, S. Effects of microplastics on soil properties: current knowledge and future perspectives. *J. Hazard. Mater.* **424**, 127531 (2022). 34740160 10.1016/j.jhazmat.2021.127531

[123] Roy, T., Dey, T. K. & Jamal, M. Microplastic/nanoplastic toxicity in plants: an imminent concern. *Environ. Monit. Assess.* **195**, 27 (2023). 10.1007/s10661-022-10654-z PMC9589797 36279030

[124] Larue, C., Sarret, G., Castillo‐Michel, H. & Pradas Del Real, A. E. A critical review on the impacts of nanoplastics and microplastics on aquatic and terrestrial photosynthetic organisms. *Small* **17**, 2005834 (2021). 10.1002/smll.202005834 33811450

[125] Lozano, Y. M. & Rillig, M. C. Effects of microplastic fibers and drought on plant communities. *Environ. Sci. Technol.* **54**, 6166–6173 (2020). 32289223 10.1021/acs.est.0c01051 PMC7241422

[126] Klaus, F., Tscharntke, T., Bischoff, G. & Grass, I. Floral resource diversification promotes solitary bee reproduction and may offset insecticide effects – evidence from a semi‐field experiment. *Ecol. Lett.* **24**, 668–675 (2021). 33524201 10.1111/ele.13683

[127] Mehrabi, Z. et al. Research priorities for global food security under extreme events. *One Earth* **5**, 756–766 (2022). 35898653 10.1016/j.oneear.2022.06.008 PMC9307291

[128] Garibaldi, L. A. et al. Wild pollinators enhance fruit set of crops regardless of honey bee abundance. *Science* **339**, 1608–1611 (2013). 23449997 10.1126/science.1230200

[129] Geyer, R., Jambeck, J. R. & Law, K. L. Production, use, and fate of all plastics ever made. *Sci. Adv.* **3**, e1700782 (2017). 28776036 10.1126/sciadv.1700782 PMC5517107

[130] Wheeler, T. & Von Braun, J. Climate change impacts on global food security. *Science* **341**, 508–513 (2013). 23908229 10.1126/science.1239402

[131] UN. *Kunming-Montreal Global Biodiversity Framework*. https://www.cbd.int/doc/decisions/cop-15/cop-15-dec-04-en.pdf (2022).

[132] Estrada-Carmona, N., Sánchez, A. C., Remans, R. & Jones, S. K. Complex agricultural landscapes host more biodiversity than simple ones: A global meta-analysis. *Proc. Natl Acad. Sci. USA* **119**, e2203385119 (2022). 36095174 10.1073/pnas.2203385119 PMC9499564

[133] Renard, D. & Tilman, D. National food production stabilized by crop diversity. *Nature* **571**, 257–260 (2019). 31217589 10.1038/s41586-019-1316-y

[134] Jing, S. et al. Non-destructive extraction and separation of nano- and microplastics from environmental samples by density gradient ultracentrifugation. *Anal. Chem.* **94**, 15280–15287 (2022). 36278923 10.1021/acs.analchem.2c02543

[135] Okoffo, E. D. et al. Release of plastics to Australian land from biosolids end-use. *Environ. Sci. Technol.* **54**, 15132–15141 (2020). 33200922 10.1021/acs.est.0c05867

[136] Proesmans, W. et al. Pathways for novel epidemiology: plant–pollinator–pathogen networks and global change. *Trends Ecol. Evol.* **36**, 623–636 (2021). 33865639 10.1016/j.tree.2021.03.006

[137] Abhijith, K. V. et al. Air pollution abatement performances of green infrastructure in open road and built-up street canyon environments – A review. *Atmos. Environ.* **162**, 71–86 (2017).

[138] Willis, K. J. & Petrokofsky, G. The natural capital of city trees. *Science* **356**, 374–376 (2017). 28450596 10.1126/science.aam9724

[139] Barwise, Y. & Kumar, P. Designing vegetation barriers for urban air pollution abatement: a practical review for appropriate plant species selection. *Npj Clim. Atmos. Sci.* **3**, 12 (2020).

[140] Ottosen, T.-B. & Kumar, P. The influence of the vegetation cycle on the mitigation of air pollution by a deciduous roadside hedge. *Sustain. Cities Soc.* **53**, 101919 (2020).

[141] Villarreal-Rosas, J. et al. Advancing systematic conservation planning for ecosystem services. *Trends Ecol. Evol.* **35**, 1129–1139 (2020). 32977982 10.1016/j.tree.2020.08.016

