# Supplementary material for: Plastic pollution in agricultural landscapes: an overlooked threat to pollination, biocontrol and food security
Source: Nat Commun. 2024 Sep 28;15:8413. doi: 10.1038/s41467-024-52734-3 (PMC11437009; doi:10.1038/s41467-024-52734-3)
Supplement: Supplementary file 1 — Supplementary Information [file 41467_2024_52734_MOESM1_ESM.pdf]

SUPPLEMENTARY MATERIAL

Plastic pollution in agricultural landscapes: an overlooked threat to pollination, biocontrol and food security

Dong Sheng[1,2,3,$], Siyuan Jing[1,2,4,$], Xueqing He[1,5], Alexandra-Maria Klein[6], Heinz-R. Köhler[7], Thomas C. Wanger[1,2,8,*]

1. Sustainable Agricultural Systems & Engineering Lab, School of Engineering, Westlake University, Hangzhou 310030, China
2. Key Laboratory of Coastal Environment and Resources of Zhejiang Province, School of Engineering, Westlake University, Hangzhou 310024, China
3. College of Environmental and Resource Sciences, Zhejiang University, Hangzhou 310030, China
4. Department of Environmental Science and Engineering, Fudan University, Shanghai 200438, China
5. Department of Health and Environmental Sciences, School of Science, Xi’an Jiaotong-Liverpool University; Suzhou 215123, China
6. Nature Conservation and Landscape Ecology, University of Freiburg, Freiburg 79106, Germany
7. Animal Physiological Ecology, University of Tübingen, Tübingen 72076, Germany
8. Agroecology, University of Göttingen, Göttingen 37073, Germany

* Corresponding author: tomcwanger@gmail.com (TCW)

[$] contributed equally

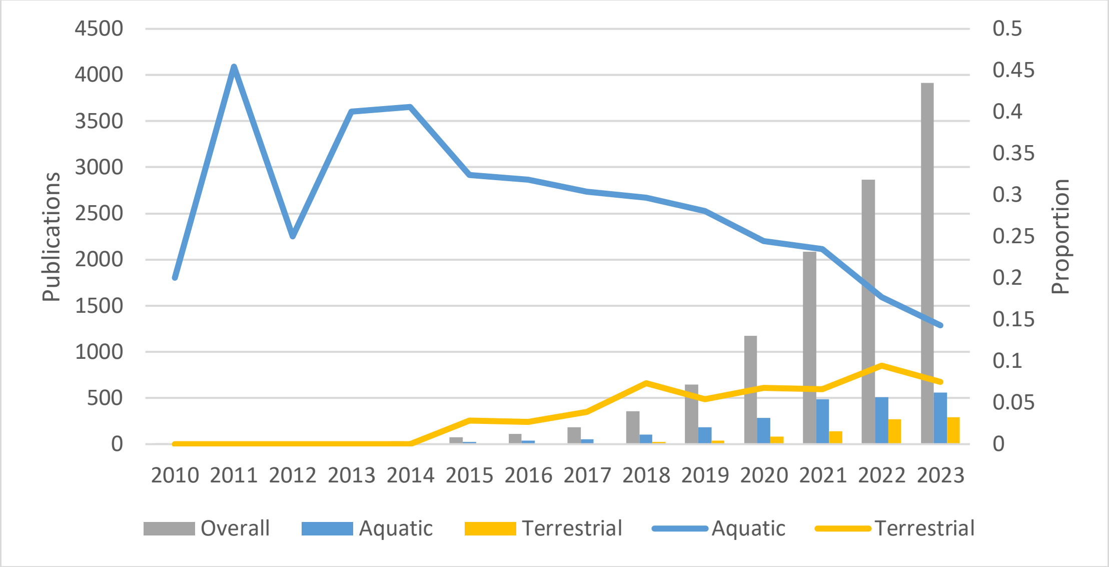

Figure 1. The trends of publications about NMP since 2010s. Grey/blue/yellow bars represent number of publications on overall, aquatic, and terrestrial NMP studies, respectively. Blue/yellow lines depict the proportion of aquatic/terrestrial system-related NMP studies. This figure is based on a Web of Science search on April 30th, 2024 with the following search string: “AK = (microplastic* OR nanoplastic*)”, “AK = ((microplastic* OR nanoplastic*) AND (aquati* OR marine* OR water* OR river* OR lake*))”, “AK = ((microplastic* OR nanoplastic*) AND (terre* OR edaph* OR soil* OR land* OR field*))”, respectively.

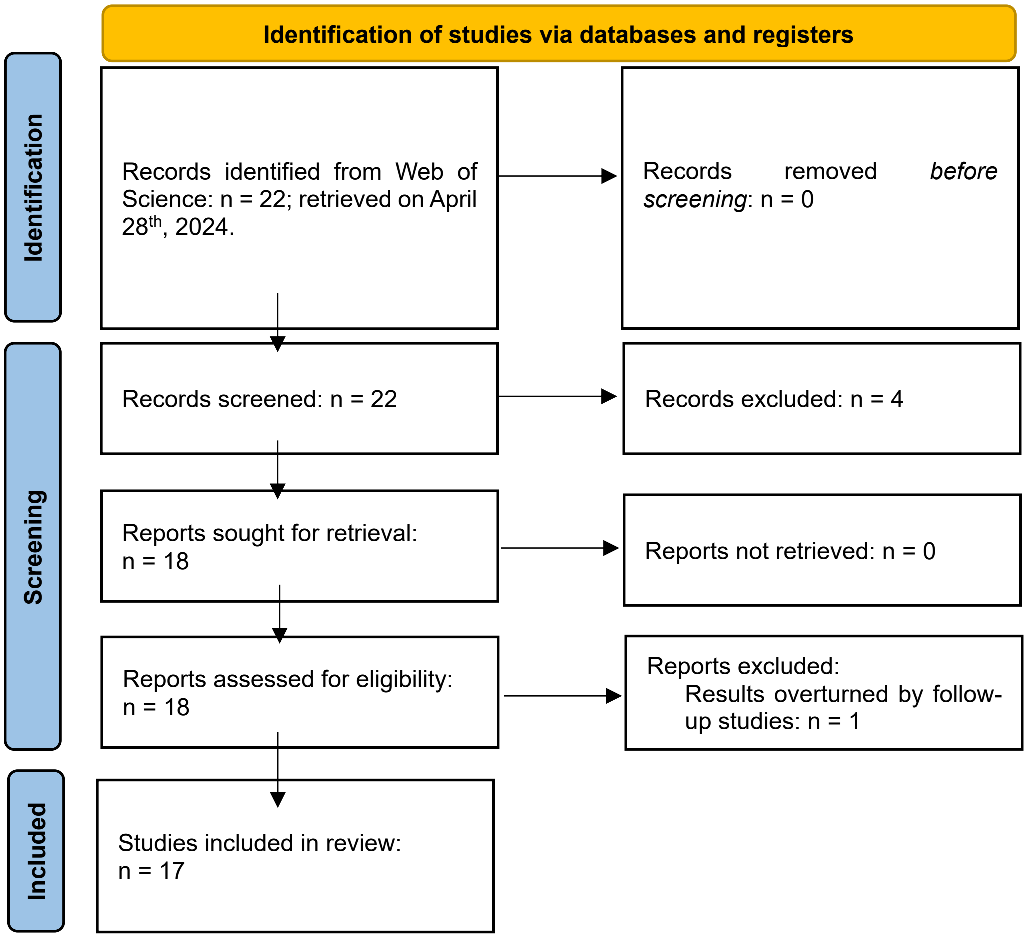

Figure 2. The PRISMA Chart of the systematic literature review Nano/Microplastic effects on pollination services.

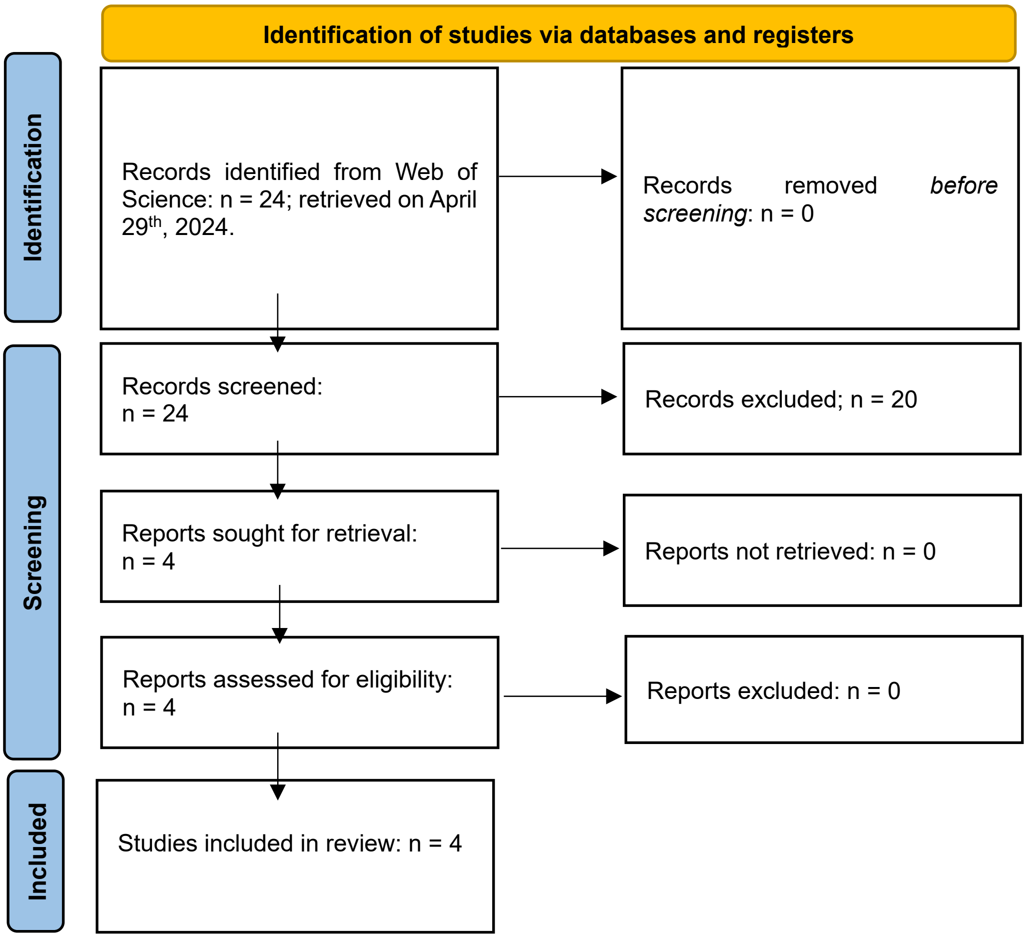

Figure 3. The PRISMA Chart of the systematic literature review Nano/Microplastic effects on biological pest control services.

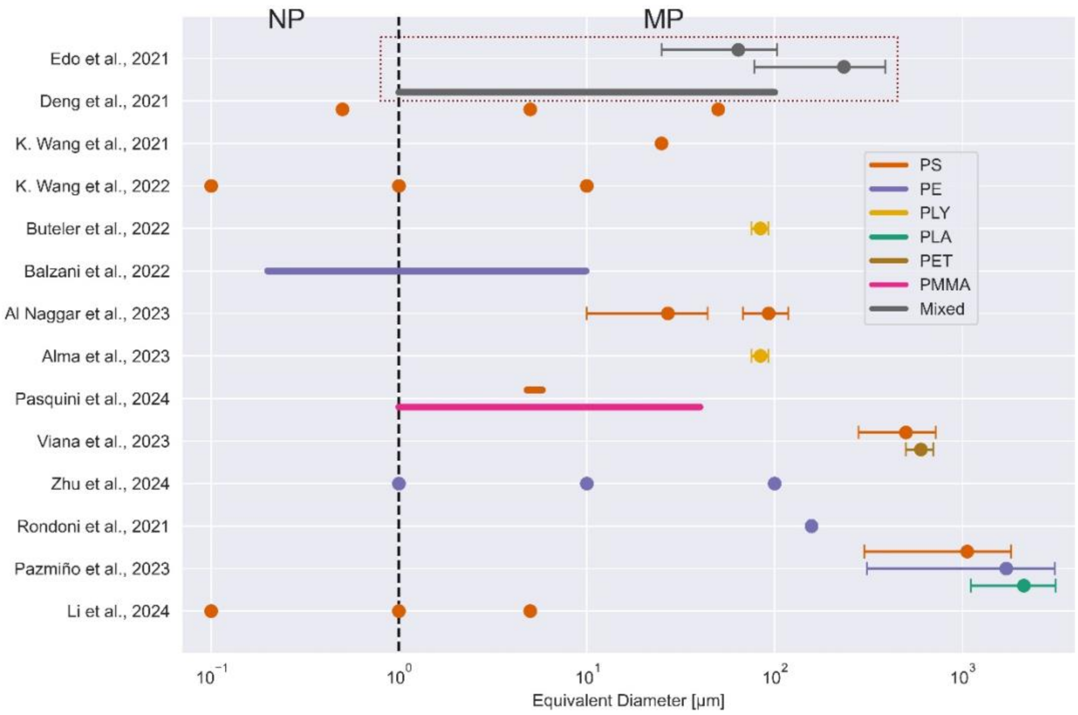

Figure 4: Equivalent diameters of NMP from literature, including field investigations and experiment setups. Scatters with error bars represent mean diameters ± standard errors, and lines represent diameter spans (minima ~ maxima). The red dotted rectangle represents field investigation data, while the rest are lab experiments. Equivalent diameters of fiber-shaped particles were calculated as aerodynamic diameters.[1] Overall, current experiments varied in the NMP types, diameters, and doses across lab and field experiments. Thus, with the current available studies, it is difficult to understand real implications of plastic pollution on pollinators and pest control agents. Abbreviations: NP = nanoplastics, MP = microplastics, PE = polyethylene, PS = polystyrene, PLY = polyester, PLA = polylactic acid, PET = polyethylene terephthalate, PMMA = polymethyl methacrylate.

1. Prodi, V., De Zaiacomo, T., Hochrainer, D. & Spurny, K. Fibre collection and measurement with the inertial spectrometer. *J. Aerosol Sci.* **13**, 49–58 (1982).
